# Exploring Roles of Stakeholders in Combating Substance Abuse in the DIMAMO Surveillance Site, South Africa

**DOI:** 10.1177/11782218221147498

**Published:** 2023-02-27

**Authors:** Livhuwani Muthelo, Masenyani Oupa Mbombi, Peter Mphekgwana, Linneth Nkateko Mabila, Inos Dhau, Joseph Tlouyamma, Rathani Nemuramba, Reneilwe Given Mashaba, Katlego Mothapo, Cairo Bruce Ntimana, Eric Maimela

**Affiliations:** 1 Department of Nursing Science, University of Limpopo, Polokwane, South Africa; 2 Research Administration and Development, University of Limpopo, Polokwane, South Africa; 3 Department of Pharmacy, University of Limpopo, Polokwane, South Africa; 4 Department of Geography and Environmental Studies, University of Limpopo, Polokwane, South Africa; 5 Department of Computer Science, University of Limpopo, Polokwane, South Africa; 6 DIMAMO Population Health Research Centre, University of Limpopo, Polokwane, South Africa; 7 Department of Public Health, University of Limpopo, Polokwane, South Africa

**Keywords:** Prevalence, substance abuse, DIMAMO, stakeholders

## Abstract

**Background:**

The increasing prevalence of substance abuse in rural areas of Limpopo Province is a concern for most stakeholders including the families, South Africa Police Service, and social workers. Combating Substance Abuse requires the active roles of various stakeholders in the rural community, due to limited resources for prevention, treatment, and recovery.

**Purpose:**

To report on the roles of stakeholders in tackling Substance Abuse during the awareness campaign conducted in the deep rural community of Limpopo Province, DIMAMO surveillance area.

**Methods:**

Qualitative narrative design was adopted to explore the roles of stakeholders in combating Substance Abuse during the awareness campaign conducted in the deep rural community. The population consisted of different stakeholders who play an active role in reducing Substance Abuse. The triangulation method was used for data collection (interviews, observations, and taking field notes during presentations). Purposive sampling was used to select all the available stakeholders who actively combat substance abuse in the communities. Thematic narrative analysis was used to analyze the interviews conducted with and content presented by the stakeholders to generate the themes.

**Results:**

The prevalence of Substance Abuse among the youth in the Dikgale community is high with a growing trend of Crystal Meth, “nyaope,” and Cannabis(marijuana). The prevalence is worsened by the diverse challenges experienced by the families and stakeholders which impact the strategies targeted to combat Substance Abuse.

**Conclusion:**

The findings indicated the need for strong collaborations among the stakeholders (including school leadership) to successfully combat Substance Abuse in rural areas. The findings demonstrated a need for a well-capacitated healthcare services with adequate rehabilitation centers and well-trained healthcare providers for combating Substance Abuse to minimize victim stigmatization.

## Introduction and Background

Substance abuse is when an individual excessively uses psychoactive drugs, such as alcohol, prescribed, or illegal drugs.^1^ Worldwide, substance abuse has been reported as a major challenge, with an estimated 15.3 million of the population suffering from substance abuse and 3.3 million deaths annually.^2^ The use of psychoactive substances such as alcohol, cigarettes, marijuana, cocaine, heroin, and the glue has been increasing since they are readily available and affordable in their geographical locations.^3^ Exposure to substances, affordability, and peer pressure are the prevailing factors increasing substance abuse among youth.^4^ In addition, about 16.0% of substance users aged 15 years and older engage in heavy episodic drinking globally for various reasons.^5^ For instance, prior studies have documented a strong association between unemployment and problematic substance abuse among the age group 15 years and above, including alcohol, marijuana, and illicit drugs. The prevalence of substance abuse is associated with unemployment, coupled with poverty, low educational status, poor access to mental healthcare, driving while intoxicated, and pattern of adverse social concerns, such as failure to meet work, family, or school obligations, interpersonal conflicts, or violence while under the influence was common among rural Americans.^5^ In comparison, the overall prevalence of substance abuse in sub-Saharan Africa was 41.6%, with the highest rate in Central Africa at 55.5%.^6^ Cannabis was reported as the most illicit drug with the highest prevalence reported in Central and West Africa, with rates ranging between 5.2% and 13.5%, 7% in both urban and rural areas.^7^ In South Africa, Sommer et al^8^ noted Alcohol abuse as the most common form of substance abuse in rural areas, followed by the misuse of illicit drugs such as Cannabis, Methamphetamine and Methaqualone compared to Central and Western countries Africa. Additionally, in SA Cannabis has been identified as one of the most highly used drug with around 3.7% of the population using Cannabis, this is followed by cocaine (1%), amphetamines (1%), opioids (0.5%), opiates (0.4%), ecstasy-type drugs (0.3%), and prescribed opiates (0.1%).^8^ Young people started drinking alcohol and using drugs at an early age since they had access to these substances at home, from friends, in shops, and from street vendors^4^ So far, South Africa seems to be the only African country that reported the association between substance abuse and poverty, unemployment, and criminal activities with diverse impacts on families and communities.^9,10^ The consequences of substance abuse on individuals, societies, and families include health, psychological, social, and economic burdens^11,12^ Furthermore, excessive consumption of substances increases the odds of criminal activities, aggressive and risky sexual behavior, personality disorders, drug dependency, increased school drop-out rate, increased risk of death from overdose and suicide and poor academic performance.^12,13^ Moreover, Mokwena and Huma (2014) noted that 15% of South Africans have drug-related problems that disrupt and complicate the daily lives of both the affected and the users.^14^ Thus, substance abuse represents a major public health and socio-economic problem, especially among adolescents.^14,15^ Mothibi^16^ states that high-rate alcohol abuse has been estimated to have attributed to between 65%and 70% of violent crimes in Cape Town. World Health Organisation (WHO)^7^ posits that there is a need for treatment and rehabilitation facilities, especially in low- and middle-income countries. Substance abuse has reached epidemic proportions, hence intervention research is needed to develop effective methods of preventing and treating the harms associated with substance abuse.^16^ Various strategies have been widely discussed, including organizing awareness campaigns, providing drug information and counseling, and defining the role of rural communities and stakeholders in preventing substance abuse.^5,17^ Other psychosocial intervention approaches identified include 3 multi-component risk reduction interventions, including alcohol/HIV risk reduction interventions.^18^ Substance abuse can be hard to combat, especially in rural areas due to limited resources for prevention, treatment, and recovery. In a recent study done by Mphekgwana et al,^19^ the DIMAMO population group, the prevalence of substance abuse was found to be high with alcohol use being (83%) and smoking (86%). The fight against substance abuse can be effective with the involvement of different community stakeholders and the proper allocation of required resources such as awareness campaigns.^4^ According to the South African Government,^20^ awareness campaigns such as the Anti-Substance Abuse Program of Action are perfect examples of primary prevention strategies that build a community free of Alcohol and Drug Abuse. Although Mphekgwana et al^19^ noted a high prevalence of substance abuse, very little was done to intervene in the substance abuse in the Dikgale Community. In 2022, the DIMAMO surveillance unit organized a substance abuse awareness campaign with key stakeholders to educate the Dikgale community about the risk associated with substance and available services to combat substance abuse. Hence, this paper aims to report on the role of stakeholders in tackling substance abuse during the awareness campaign conducted in the deep rural community of Limpopo Province, DIMAMO surveillance area, using a narrative qualitative design.

## Material and Methods

### Study design

The qualitative narrative design was found suitable to narrate integrated services to combat substance abuse by the key stakeholders. Maxwell^21^ posits that qualitative research work with the universe of meanings, motives, aspirations, beliefs, values, and attitudes, which corresponds to a deeper space of relationships, processes, and phenomena. The design provided an opportunity for the key stakeholders to describe their roles in combating substance abuse, incorporating their motives and aspirations. The use of qualitative narrative design enabled the authors to narrate the roles of the stakeholders on Substance Abuse as presented during the awareness campaign and interviews. Moreover, the use of narrative design deepened the authors’ understanding of various dimensions of Substance Abuse, as illustrated using the triangulation method of data collection.

### Population and sampling

The population consisted of 20 key stakeholders who are actively participating in Substance Abuse within communities. These included 3 South African Police Services (SAPS) representatives in a local area, 3 members of the South African National Council on Alcoholism and Drug Dependence (SANCA) at a provincial level, 1 social worker and a professional nurse from Dikgale Clinic, 1 ward councilor as a political structure, 4 Community Advisory Team (CAT) within Dikgale area, and 3 Youth Desk/dialoqoue from the Limpopo Provincial office. Green and Thorogood (2014), posit that a representative sample is necessary for generalizing the results across the entire population.^22^ Purposive sampling was used to select the key stakeholders for participation in substance awareness. The adoption of purposive sampling allowed the authors to select stakeholders who had insight into substance abuse-related factors and the ways to overcome them. The DIMAMO community engagement officer recruited the key stakeholders through the assistance of the Community Advisory Team (CAT) leaders. Therefore, the selection of the stakeholders was based on their roles for combating Substance Abuse in rural areas.

### Data collection

To obtain broader and more detailed information about the key roles of stakeholders’ services in combating Substance Abuse, the authors adopted a triangulation approach for data collection which included observations, interviews, and taking of field notes. For instance, the authors used the observation method to listen to and observe the services provided by different key stakeholders during the awareness day. To supplement the observations, the authors collected pamphlets from other stakeholders and noted their key aspects as field notes. To explore more about the services of the stakeholders, semi-structured one-on-one interviews, which lasted for about 30 to 45 minutes, were conducted in a private room by the 2 primary authors with each member of the stakeholders. The primary authors explained the purpose of performing the interviews, the voluntary nature of participation, and the freedom to withdraw at any time before the interviews were conducted. The quality of data collected was verified and enhanced by 5 authors, triangulation methods for data collection, and literature support about Substance Abuse (Table 1).

**Table 1.** Interview guide.

Central question
Kindly describe your role in the prevention and management of substance abuse within the DIMAMO community
Probing questions
✓ What are the challenges that you experience during the implementation of the activities to prevent substance abuse?
✓ What is the prevalence of substance abuse in the DIMAMO community?
✓ How is the relationship with other stakeholders in collaboration to reduce substance abuse?
✓ What are you think can be further done to overcome the current state of substance abuse within DIMAMO community?

### Data analysis

Since the paper adopted narrative design, the authors used structural thematic and visual narrative qualitative analysis. For example, thematic narrative analysis was used to analyze the content presented by the stakeholders as transcripts from the data. This content was used to generate the themes. Two primary authors who read through the transcribed text several times analyzed data to familiarize themselves with the data. After that, data was broken down into smaller meaning units using a deductive approach (pre-identified codes) to answer the research question, which was set out in the aim of the study. An inductive approach (emerging data) was also used to develop codes guided by field notes and the transcribed data.^23^ Furthermore, the inductive approach assisted the researchers in reflecting on the data in new ways, making it easier to identify connections between meaningful words. Erlingsson and Brysiewicz (2017) posit that the codes created inductively may change as the study progresses and more data become available.^24^ Therefore, the coding process was performed repeatedly, starting on different pages of the text each time by both primary researchers until the themes emerged. To increase the stability and reliability, the 2 primary authors exchanged the transcripts and the coding structure to verify the identified themes and how they were interpreted. This was followed by a meeting with the experienced qualitative researchers where a comparison was made, and an agreement on the themes was reached. The themes were arranged following a structural approach to clearly narrate the Dikgale community’s awareness campaign. To supplement the story’s structure, authors adopted visual narrative analysis encompassing words and images from the pamphlets that the key stakeholders distributed.

## Results

### Context of the awareness

Substance abuse awareness was conducted on the 21st of May 2022 at Ramabu High School (Dikgale Village) within Capricorn District of Limpopo Province in South Africa. Dikgale Village is one of the villages catered to within the DIMAMO surveillance units. DIMAMO site is located in rural areas about 30 km North of Polokwane. In this population, the prevalence of substance abuse was identified to be a challenge, with 86% smokers and 83% abusing alcohol.^19^ This study was conducted as part of the South African Population Research Infrastructure Network (SAPRIN), an initiative hosted by the South African Medical Research Council and receives long-term from the National Department of Science and Innovation.

Table 2 gives the demographic traits of the participants. The majority of the stakeholders were the community advisory team members who are local members which suggest how they value the services targeted toward combating substance abuse in the community. The study was constituted by 2 members from the Department of Health and Social Services who described the role of nurses and social workers in combating substance abuse. Since the study targeted combating substance amongst young people, 3 members from the youth desk and dialog presented the roles of youth in dealing with substance abuse. The age range of stakeholders was between 21 and 65 years which indicates the significant involvement of different age group member both local and provincial stakeholders as services toward combating substance abuse.

**Table 2.** Demographics of key stakeholders.

Category stakeholder	Gender	Age (years)	Workplace coverage	Total
South African Police Service [SAPS] (two senior members—commissioners & 1 warrant officer)	Male	31-40	Local police	2
	Male	51-60	Provincial police	1
South African National Council on Alcoholism and drug Dependence (SANCA)’ members	Male	31-40	Provincial office	3
Social worker	Female	31-40	Local clinic	1
Professional nurse	Female	41-50	Local clinic	1
Ward counselor	Female	41-50	Local political structure	1
Community advisory council	Male	51-60	Local members	3
	Female	41-50	Local member	1
Youth desk and dialog	Male	20-30	Limpopo provincial office Local area	3
Polokwane Football Association (POLFA)	Male	41-50	Provincial Football Association	1
Turfloop (TURF) FM	Male	31-40	Local radio station	1

### Findings from observations and semi-structured interviews

The section below presents the results from the unstructured observations and semi-structured interviews.

#### Unstructured observations

We noted the significant roles played by stakeholders in combating substance abuse from the presentations and the printed pamphlets. For instance, the pamphlets outline the services provided by the stakeholders such as rehabilitation and counseling as indicated in the SANCA pamphlet. Some of the roles included recreational activities such as soccer tournaments and aerobics which encourage young people to stay away from substances.

***SAPS*** *presented one of the activities as “we educate young people about types of substances and the consequences of their use, by conducting Roadblocks with pamphlets to create awareness about SA.”*

**Figure:**
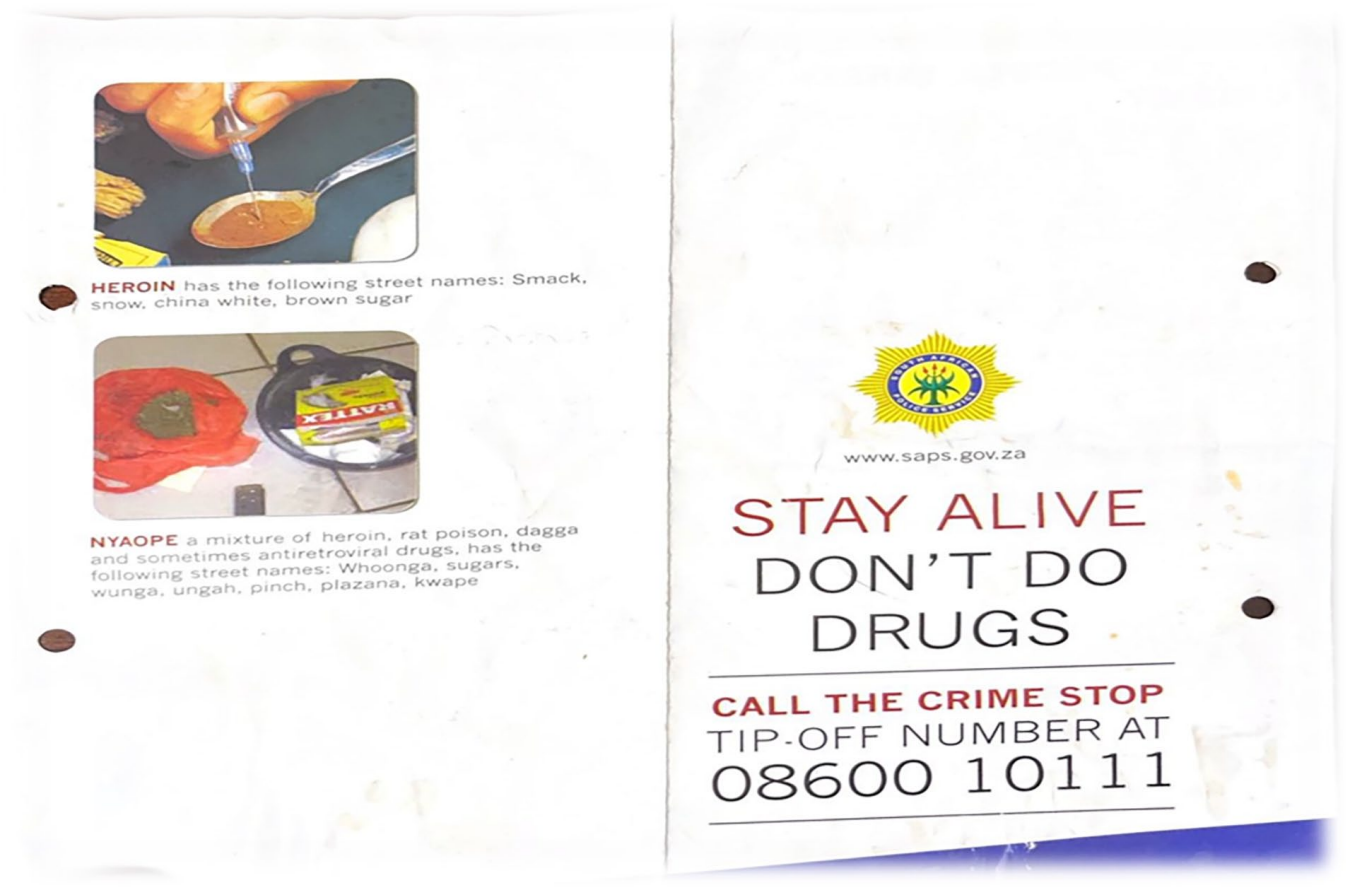


SANCA shared some of its activities, including “providing education to the community about services to reduce Substance Abuse (SA) and conduct community awareness about the treatment of SA and provide treatment to people who abuse substance.”

**Figure:**
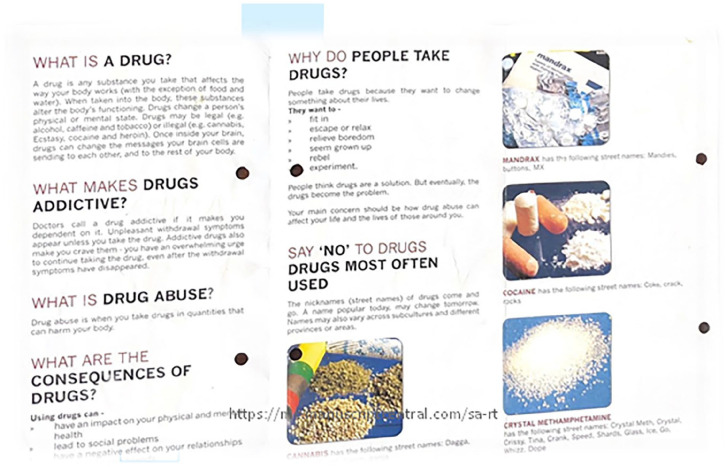


Some stakeholders indicated that their role involves youth empowerment to combat substance abuse. The findings are demonstrated below with the illustrations:

Community Advisory Team (CAT) alluded during their presentation that they assist and emphasize to young people the importance of youth and their roles in the community while ensuring that they don`t get involved in Substance Abuse (SA).

While Youth Desk and Dialog presented some of their roles as “encouraging young people to unite and fight SA and assist them in dealing with SA by engaging them in recreational activities like soccer, drama, and agricultural activities to address health problems associated with SA while encouraging them to abstain from abusing the substance.

These observation findings were supplemented by interviewing the key stakeholders with a semi-structured interview guide (see the attached file). The use of an interview guide assisted the researchers to solicit the narrative and uniformity of the questions during the interview process.^23^

#### Semi-structured one-on-one interviews

##### Theme 1: Roles and activities of stakeholders in combating substance abuse

Stakeholders shared different roles and activities for combating substance abuse during their presentations.

##### Sub-theme 1.1: Description of the stakeholder’s educational role and campaigns

To fight against substance abuse, stakeholders reported that they provide education to the community about services to reduce Substance Abuse (SA). The educational activities are achieved through door-to-door campaigns in the community and doing school visits to address youth about the use of substances. For example, below are some of the extracts

SAPS **“***We do door-to-door campaigns to the community and school visits to address youth about the use of substances”*

In addition, another participant from the provincial SAPS added by saying “*We Conduct Roadblocks with pamphlets to create awareness about SA”*

**Social worker** *“We Conduct campaigns for the prevention of SA & inform those who abuse substances about the dangers of it.”*

**SANCA** *“We conduct campaigns for the community about services to reduce SA. We also conduct school visits and do annual campaigns about SA.”*

##### Sub-theme 1.2: Diverse roles regarding treatment and management of SA

Different stakeholders also shared their roles and activities regarding treatment for those abusing substances. Some of these roles include referring people to tertiary health facilities for treatment, rehabilitation, and provision of counseling to the person, families, and even the community at large.

**SANCA”** *we provide treatment for people who abuse substances and community awareness about the treatment of SA”*

**Social worker:** *we provide counselling to those who abuse substances in the facilities for further management and rehabilitation purposes.*

##### Sub-theme 1.3: Explanation of different roles through youth empowerment

Some stakeholders indicated that their role involves youth empowerment to combat substance abuse. The findings are demonstrated below with the illustrations:

**Community Advisory Team (CAT)** *“We emphasize the importance of youth in the community while ensuring that they don’t get involved in SA. We also recognize recreational activities such as soccer, netball & aerobics.”*

**Youth Desk Dialogue ‘***To encourage young people to unite and fight SA”. We also empower them by engaging them in recreational activities like soccer, drama, and agricultural activities.*

All presentations and interviews illustrated that these stakeholders’ roles and activities in reducing substance abuse are achieved by working with other stakeholders or collaborators at a local and provincial level.

##### Theme 2: Outlining the importance of service collaborations for reducing Substance Abuse at the community level

**Figure 1.**
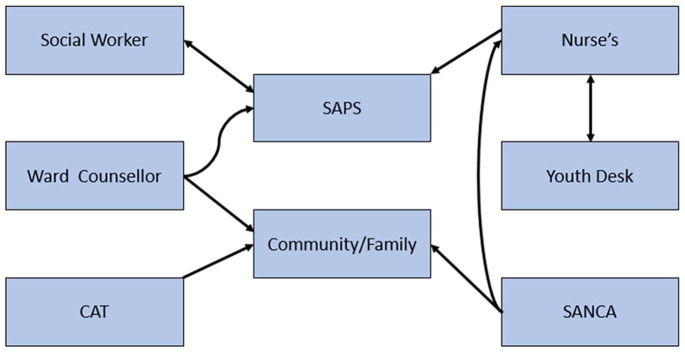
Service collaborations by stakeholders.

Different stakeholders outlined that they achieve their scope of services regarding reducing substance abuse by working with collaborators at a local and provincial level. The findings demonstrated the need to work together with key stakeholders to reduce SA. As depicted in Figure 1 below, stakeholders’ most common desired services include collaborations with family members (parents included), South African Police Service (SAPS) social worker, The Community Advisory Team (CAT), the youth desk, the South African National Council on Alcoholism and Drug Dependence (SANCA)’ and medical services to reduce substance abuse and its complications.

Figure 1 above further indicates that the family of the person abusing substances and SAPS South African Police Service (SAPS) were the centers of collaborated services for combating substance abuse

##### Sub-theme 2.1: Reporting of collaboration through referrals

The primary reason for collaboration is mainly to assist the person with the family/community; hence it is regarded as a center of collaboration. The illustration below supports the need for collaboration.

**SAPS** *“We refer the young people from the community with challenges of SA to Employee Health wellness welfare that engages with social workers and by involving parents to support & avoid judging them.”*

***SANCA*** *“We refer to Substance abusers for rehabilitation outside Dikgale. Also, we collaborate with SAPS for police-related services.”*

*As a community Advisory Team, “we refer youth to be rehabilitated with sports activities to Polokwane Football Association which draw away the attention of youths from SA”*

##### Sub-theme 2.2: Narrative on the importance of working relationships amongst stakeholders

On the other hand, some stakeholders narrated that the success of dealing with substance abusers are grounded on the working relationship between the South African Police Services and healthcare rehabilitation centers. For example, the social worker reported that;

**Nursing Service** “We *mostly collaborate with the youth desk, social workers, SAPS, and Community/families”*

**Social workers** *“We collaborate with SAPS, and engage with school principals for youth abusing substances, and refer them to rehabilitation centers outside Dikgale village.” Social workers further provided reasons for referring young people to SAPS as the consistent abuse of most dangerous substances has intensified within Dikgale village.*

##### Theme 3: The Prevalence of certain substances being abused in the community

Most of the stakeholders indicated a concern regarding the growing trends of some dangerous substances abused within DIMAMO community. Stakeholders reported a need for integrated services targeted to reduce this ever-increasing trend of Crystal Meth that is spreading at a high level among young people in the community and among school kids. Some other substances listed include abuse of alcohol, “Nyaope,” and *Cannabis (Marijuana) which is notably high in the community*. The need to urgently design integrated services for Substance Abuse is exacerbated by the diverse challenges brought by substance abusers to their families and communities.

##### Theme 4: Diverse challenges to the families and stakeholders regarding services for combating substance abuse

There are diverse challenges of substance abuse that impact families and stakeholders’ services. The challenges are categorized as family-related and services related with some stakeholders demonstrating that SA has intensified challenges experienced by families of substance abusers.

The stakeholders reported the following regarding family-related challenges:

**SAPS: *“****Parents’ conflicts/violence perpetuates SA as a coping strategy.”*

***Social worker “****Most men don`t accept that they abuse substances with the intent to protect their religious organizations”*

##### Sub-theme 4.2: Diverse challenges related to poor access to rehabilitation centers

Stakeholders expressed concern regarding access to adequate rehabilitation centers, promotional materials, and effective counseling sessions with substance abusers.

**SANCA and Social worker** *“The rehabilitation centres with limited capacity”*

**SANCA** *“The stigmatization of the referred clients by healthcare providers, and doctors not having adequate resources (for drug de-toxication), contribute to a slow success rate due to relapse challenges.”*

They have limited promotional materials to motivate people to stop SA (SAPS). Additionally, other stakeholders expressed concern regarding the expected collaborative services from different stakeholders. For instance, socially expected support services from SAPS, while SANCA demonstrated that the schools do not play an active role when expected. Below are examples of some of the stakeholder’s extractions:

**Social worker** *“the challenge includes the local SAPS not actively involved”*

**SANCA** “*School visit challenges include language barriers and a lack of understanding of the scope of SANCA by school leadership.”*

These challenges include increased family violence, conflicts, divorce, rape, school dropouts, and increased unemployment for those young people who have completed the course of rehabilitation. stakeholders expressed various ways to address some of these challenges.

##### THEME 5: Recommendations for improving services to reduce SA

The stakeholders indicated the need for integrated support services to reduce substance abuse in the community. The collaborations include working with universities to assist in the rehabilitation process, basic schools, and community members, and capacitating healthcare professionals to minimize stigmatization. The extracts below illustrate the recommendations for services:

**Youth dialogue:** *“To collaborate with the basic schools and universities to assist those who youth in completing their training; community members, and SAPS in fighting SA.*

**SANCA:** *“Capacitate healthcare providers to minimize stigmatization, and provision adequate resources, and enhance the collaboration with SAPS for search warrants of substances in school/families, social workers, and school leadership for a successful campaigns.”*

## Discussion

This project aimed to explore the underlying risk factors contributing to substance abuse, the nature of the substance they use, and integrated strategies that have been used to reduce substance abuse in the Dikgale community. The study findings highlight the importance of stakeholders’ collaborations and sustaining the existing ones for reducing substance abuse at the community level. The stakeholders’ involvement in substance abuse awareness in combating SA within Dikgale successfully demonstrated a need to enforce and sustain the collaborations. This follows the common primary activities and roles of the stakeholders in reducing substance abuse. Palombi et al^25^ study on Community Forums to Address the Opioid Crisis also indicated the importance of community-integrated forums which were found to be effective in increasing overall awareness and knowledge of the opioid crisis within each community. Moreover, the forums included speakers from varied professional backgrounds, integrated cultural strengths were reported and the collaboration was highly rated in the prevention and addressing substance use with increased community member engagement.

The study further indicates the prevalence of substance abuse in the Dikgale community which is considered to be high mostly among youth. Stakeholders reported a need for integrated services targeted to reduce the growing trends of Crystal Meth including alcohol, “Nyaope,” and Cannabis (Marijuana) that are spreading at a high level among young people in the community and also among school kids. Prior studies conducted in the Dikgale area recently showed a high prevalence of alcohol use (83%) and smoking (86%) in the Dikgale community.^19^Anyanwu et al^14^ study has shown that the prevalence of substance abuse among adolescents or youth is characterized by increased adventurous tendencies and peer influences, where they start with substances like cigarettes and alcohol and gradually progress to other substances to the degree that they depend on these substances.

Considering the effect of substance abuse on individuals, societies, and families including diseases, psychological, social, and economic burdens, there is an urgent need to design and implement integrated services for substance abuse in the Dikgale community. The Surgeon General’s Report on Alcohol, Drugs, and Health^26^also outlined the impact of substance abuse as a public health concerns with associated long-term medical consequences, inclusive of substance use disorders. They further indicated the urgent need for early prevention and intervention on the misuse of substances.

The study further outlined diverse challenges to the families, community, stakeholder services due to substance abuse. Gwala^27^posits that family members are the victims of devastating effects from drug and alcohol abuse. As such they tend to experience emotional problems and often must face financial, legal, and medical implications.^27^ The most standing challenge is family violence, abuse, rape, capacitation of the healthcare professionals and community members to reduce stigmatization, and increasing the capacity of the rehabilitation centers. Sung et al^28^ raised a concern that in rural communities there is a lack of facilities to address substance abuse and that the fewer public transportation option. Our findings recommend the need to integrate, implement and sustain the existing collaborations in reducing substance abuse in the community. The collaborations among others include working with universities to assist in the rehabilitation process, basic schools, community members, and capacitating the healthcare professionals to minimize stigmatization. The findings are consistent with the study done by Pullen and Oser^18^cited the lack of interagency cooperation as a major barrier to combating substance use in urban and rural communities. Moreover, it was highlighted that effective treatment requires not only substance abuse counseling but also, a variety of other complementary services with communication among a network of facilities and providers.

## Conclusion

The paper reflected on the integrated services by the stakeholders who participated in the substance abuse awareness, which was conducted on the 21st of May 2022 in Capricorn District of Limpopo Province in South Africa. The study findings highlight the importance of integrated services to combat substance abuse in the rural community and sustain the existing collaborations in reducing substance abuse in the community. The collaborations amongst stakeholders include working with local universities and academics assisting unemployed youth with job seeking and internships to reduce the unemployment rate, basic schools, youth leaders, funding opportunities for community members, and capacitating healthcare professionals to minimize stigmatization.

## Strengths and Limitations of the Study

This was a qualitative study conducted during the DIMAMO awareness campaign in the rural areas of Limpopo Province. The strengths were identified in the use of different methods of data collection (Triangulation). The utilization of triangulation made creative ways of exploring the stakeholder’s intervention to combat substance abuse in rural communities. The limitation of the study was the qualitative nature of the study with limited participants. Therefore, the results cannot be generalized to the larger group of participants.

## References

[1] Ani GN. Prevalence of substance abuse among senior secondary students in Mainland Local Government, Lagos. Glob J Med Public Health. 2014;3:1-9.

[2] Idowu A Aremu AO Olumide A Ogunlaja AO. Substance abuse among students in selected secondary schools of an urban community of Oyo-state, South West Nigeria: implication for policy action. Afr Health Sci. 2018;18:776-785. 30603011 10.4314/ahs.v18i3.36 PMC6307013

[3] Lebese RT Ramakuela NJ Maputle MS. Perceptions of teenagers about substance abuse at Muyexe village, Mopani district of Limpopo Province, 9910 South Africa. Afr J Phys Health Edu, Recreat Dance. 2014;20:329-347.

[4] Somani S Meghani S. Substance abuse among youth: a harsh reality. Emerg Med. 2016;6:2.

[5] World Health Organization. International classification of diseases (ICD) revision, 11^th^ revision (ICD-11). WHO. 2018. Updated April 2019. Accesed May 28, 2022. https://www.who.int/classifications/icd/en/

[6] Olawole-Isaac A Ogundipe O Amoo EO Adeloye DO. Substance use among adolescents in sub-Saharan Africa: a systematic review and meta-analysis. S Afr J Child Health. 2018;12:79.

[7] WHO, Regional Office for Africa. Substance abuse. 2021. Accessed May 27, 2022. https://www.afro.who.int/health-topics/substance-abuse

[8] Sommer J Hinsberger M Elbert T , et al. The interplay between trauma, substance abuse and appetitive aggression and its relation to criminal activity among high-risk males in South Africa. Addict Behav. 2017;64:29-34. 27540760 10.1016/j.addbeh.2016.08.008 PMC5102240

[9] Stoltz E. South Africa’s longstanding drug abuse problem linked to unemployment. Mail & Guardian. 2022. Accessed June 5, 2022. https://mg.co.za

[10] Cheteni P Mah G Yohane YK. Drug-related crime and poverty in South Africa. Cogent Econ Finance. 2018;6:1-16. doi:10.1080/23322039.2018.1534528

[11] Mupara LM Tapera R Selemogwe-Matsetse M , et al. Alcohol and substance use prevention in Africa: systematic scoping review. J Subst Use. 2022;27:335-351.

[12] Nyabadza F Coetzee L. A systems dynamic model for drug abuse and drug-related crime in the Western Cape province of South Africa. Comput Math Methods Med. 2017;2017:4074197. 13. 10.1155/2017/4074197 PMC5438861 28555161

[13] Fernandes L Mokwena K. The role of locus of control in nyaope addiction treatment. S Afr Fam Pract. 2016;58:153-157.

[14] Mokwena KE Huma M . Experiences of ‘Nyaope’ users in three provinces of South Africa: substance abuse. Afr J Phys Health Educ Recreat Dance. 2014; 2:352-363.

[15] Sorsdahl K Stein DJ Corrigall J , et al. The efficacy of a blended motivational interviewing and problem solving therapy intervention to reduce substance use among patients presenting for emergency services in South Africa: a randomized controlled trial. Subst Abuse Treat Prev Policy. 2015;10:46. 26576946 10.1186/s13011-015-0042-1 PMC4650345

[16] Mothibi K. Substance abuse amongst high school learners in rural communities. Univers J Psychol. 2014;2:181-191.

[17] Machethe P Obioha E Mofokeng J. Community-based initiatives in preventing and combatting drug abuse in a South African township. Int J Res Bus Soc Sci. 2022;11:209-220.

[18] Pullen E Oser C. Barriers to substance abuse treatment in rural and urban communities: counselor perspectives. Subst Use Misuse. 2014;49:891-901. 24611820 10.3109/10826084.2014.891615 PMC3995852

[19] Mphekgwana PM Mabila LN Maimela E. Indirect and direct effects of factors associated with diabetes amongst the rural black population in the Dikgale Health and Demographic Surveillance System, South Africa. Afr J Prim Health Care Fam Med. 2021;13:2071-2936. 10.4102/phcfm.v13i1.2819 PMC8335786 34342480

[20] South African Government. Department of social development: Building a community that is free from alcohol and drug abuse. 2019. Accesed May 30, 2022. https://www.gov.za/speeches/social-development-hosts-anti-substance-abuse-campaign-exhibitions-tertiary-institutions-28

[21] Maxwell J. Qualitative Research Design: An Interactive Approach. SAGE; 2013.

[22] Green J Thorogood N. Qualitative Methods for Health Research. 3rd ed. SAGE Publications; 2014.

[23] Nowell LS Norris JM White DE Moules NJ. Thematic analysis: striving to meet the trustworthiness criteria. Int J Qual Methods. 2017;16:1-13. doi:10.1177/1609406917733847

[24] Erlingsson C Brysiewicz P . A hands-on guide to doing content analysis. Afr J Emerg Med. 2017;7:93-99. doi:10.1016/j.afjem.2017.08.001 30456117 10.1016/j.afjem.2017.08.001 PMC6234169

[25] Palombi L Olivarez M Bennett L Hawthorne AN. Community forums to address the opioid crisis: an effective grassroots approach to rural community engagement. Subst Abuse Res Treat. 2019;13:1178221819827595. 10.1177/1178221819827595 PMC6378421 30799927

[26] The Surgeon General’s Report on Alcohol, Drugs, and Health. Facing Addiction in America. Substance Abuse and Mental Health Services Administration (US); Office of the Surgeon General (US). 2016. 28252892

[27] Gwala N. Drug users: No time like the present to ‘Kick your Habit’. Health-E- News. June 2021. Accessed June 3, 2022. https://health-e.org.za

[28] Sung HE Mahoney AM Mellow J. Substance abuse treatment gap among adult parolees: prevalence, correlates, and barriers. Crim Justice Rev. 2011;36:40-57.

